# Osteoclast Giant Cell Tumor of Pancreas: A Case Report and Literature Review

**DOI:** 10.7759/cureus.4710

**Published:** 2019-05-21

**Authors:** Haisam Abid, Rosana Gnanajothy

**Affiliations:** 1 Internal Medicine, Bassett Medical Center, Cooperstown, USA; 2 Oncology, Bassett Medical Center, Cooperstown, USA

**Keywords:** pancreatic cancer, chemotherapy, osteoclast giant cell

## Abstract

We report a rare case of undifferentiated carcinoma of the pancreas with osteoclast-like giant cells (UC-OGC) in a 62-year-old female who presented with a three-month history of bilateral flank pain and significant unintentional weight loss. Computed tomography (CT) scan of the abdomen showed pancreatic tail mass, concerning for malignancy. She underwent endoscopic ultrasound (EUS) which also revealed a pancreatic mass invading into the splenic artery. CT chest and positron emission tomography (PET) scan did not reveal any metastases. The patient had a distal pancreatectomy, splenectomy, and left partial adrenalectomy. Histopathology revealed undifferentiated carcinoma of the pancreas with osteoclast-like giant cells (OGCs). The patient was recently started on adjuvant chemotherapy with capecitabine and gemcitabine and the plan is to repeat imaging to assess response. We present this case to increase clinical awareness of this rare clinical entity, and also review controversies in the management and surveillance of UC-OGC.

## Introduction

Undifferentiated carcinoma of the pancreas with osteoclast-like giant cells (UC-OGC) is an exceedingly rare exocrine tumor, that accounts for less than 1% of all pancreatic malignancies [1-2]. There are three types of giant cell tumor of the pancreas: osteoclastic, pleomorphic, and mixed; however, in 2010, the World Health Organization (WHO) grouped them together as (UC-OGC [1]. Histologically, it resembles a giant cell tumor of the bone containing osteoclastic-like multi-nucleated cells and mononuclear cells. UC-OGC has been found to have a worse prognosis, as compared to that of invasive ductal adenocarcinoma of the pancreas, because it is often found to be unresectable at the time of diagnoses due to presentation at an advanced stage, and it also tends to recur early even after complete resection. The median overall survival rate of UC-OGC is less than one year with few exceptions on literature review [2]. There have been few reports in the literature regarding the clinical, histopathological and treatment of this neoplasm. In this case report, we present a case of UC-OGC and we will also review the available literature on this rare entity.

## Case presentation

**Figure 1:**
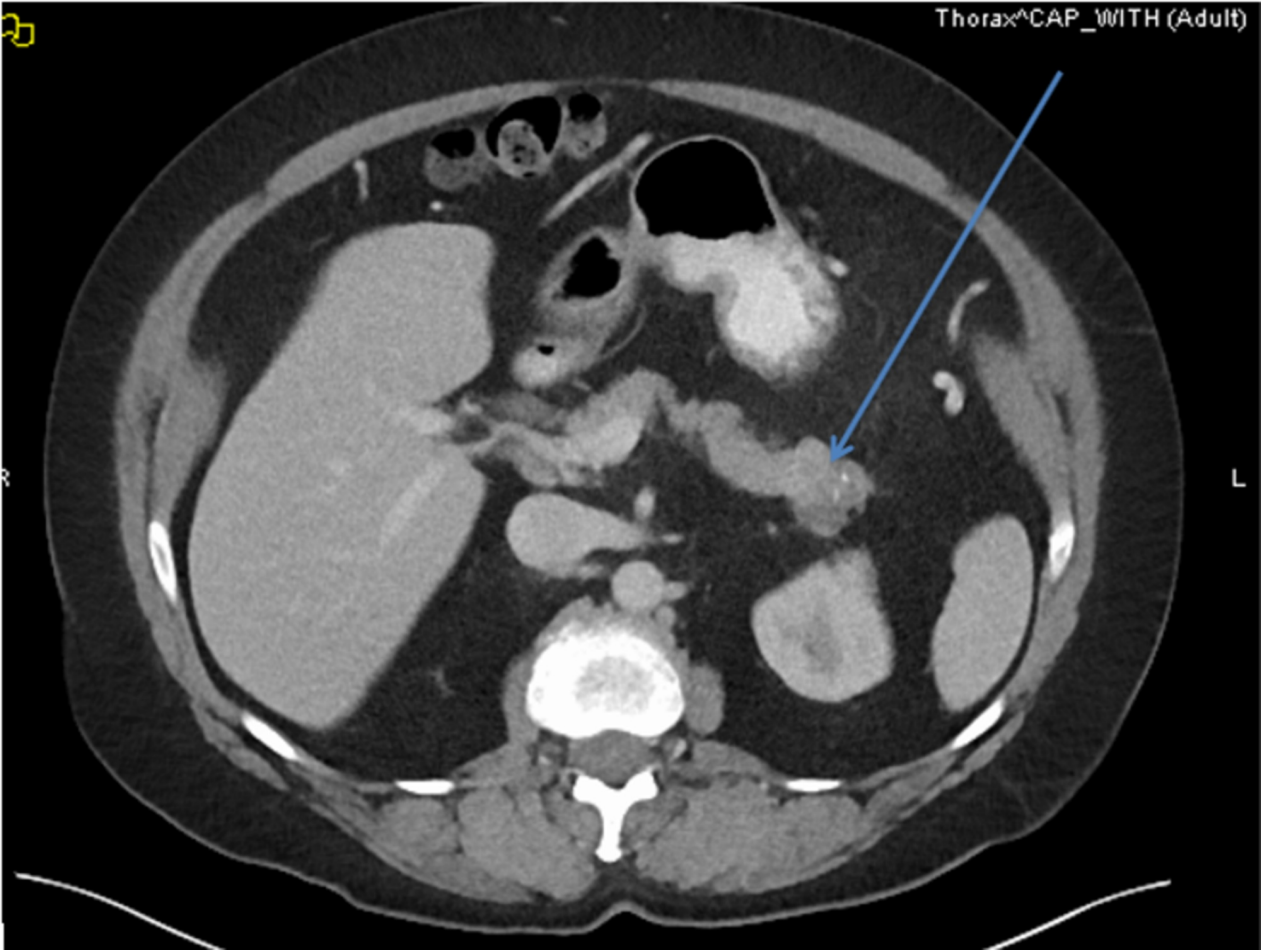
Computed tomography (CT) scan of the abdomen and pelvis with intravenous contrast showing mass in the tail of pancreas

**Figure 2:**
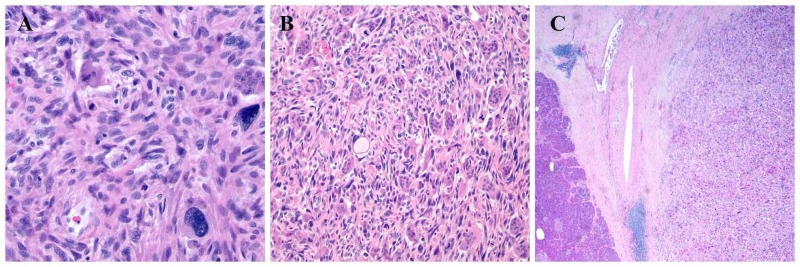
(A, B) showing particular osteoclast-like giant cells and highly pleomorphic malignant epithelial cells; (C) shows the mass expanding into pancreatic tissue

A 62-year-old female presented with a three-month history of bilateral flank pain associated with unintentional weight loss of 25 pounds. Physical examination revealed flank tenderness bilaterally without guarding or rigidity and temporal muscle wasting. Computed tomography (CT) scan of the abdomen showed a 3-centimeter (cm) mass in the pancreatic tail suspicious for malignancy associated with retroperitoneal and bilateral inguinal adenopathy without ascites (Figure 1). CT scan of the chest done did not show any metastases. Endoscopic ultrasound (EUS) revealed a 3-cm pancreatic tail mass invading the splenic artery at the superior pole of the left kidney. EUS-guided fine needle aspiration (FNA) demonstrated malignant tumor with prominent multinucleate-cell component and readily identifiable mitotic activity. Positron emission tomography (PET) scan did not show a fluorodeoxyglucose (FDG) avid lesion. The patient underwent distal pancreatectomy, splenectomy, and left partial adrenalectomy. Histopathological examination revealed UC-OGC (Figure 2). The carcinoma was associated with bone formation and extended into peri-pancreatic soft tissue. The tumor showed fibrosis and old hemorrhage, lymphovascular invasion, and had negative resection margins and no nodal metastases. The patient was started on adjuvant chemotherapy with gemcitabine and capecitabine but she did not tolerate the first cycle. She developed significant colitis. Given her significant colitis, we discontinued capecitabine and she is currently receiving gemcitabine as an adjuvant chemotherapy.

## Discussion

Pancreatic neoplasms are relatively common gastrointestinal malignancies, with adenocarcinoma of the pancreas being the second most common tumor [3]. One of the uncommon sub-types of pancreatic cancer is the undifferentiated carcinoma of the pancreas. These are very rare non-endocrine pancreatic tumor with an incidence of less than 1% of all pancreatic tumors [1-3]. In 1968, Rosai described the first case of UC-OGC. Histologically, this tumor is comprised of two cellular components: osteoclast-like giant cells (OGCs) and ovoid-to-spindle shaped mononuclear tumor cells [4].

Pancreatic UC-OGC is more common in middle-aged and elderly patients, with more than 90% of cases in patients older than age 50. The median age of diagnosis is 63 years. Females are more at risk as compared to males. Most common clinical manifestations associated with UC-OGC are upper abdominal pain and weight loss. Our patient had bilateral flank pain and significant weight loss at the time of presentation [4]. Less common clinical symptoms include loss of appetite, steatorrhea, nausea, jaundice, and anemia [5]. 

There are few case reports, which suggest the origin of undifferentiated carcinoma of the pancreas from acinar cells, mesenchymal cells, undifferentiated precursor or stem cells; however, in 2010 the World Health Organization (WHO) classification established that it originates from ductal epithelial cells and it is one of the variants of ductal adenocarcinoma of the pancreas. According to Sungho Jo, histopathological analysis of the specimen revealed the coexistence of adenocarcinoma component and undifferentiated carcinoma component with reactivity with vimentin, suggesting that the tumor originated from pancreatic ductal cells with mesenchymal differentiation [2].

The histopathological hallmark of UC-OGC is the presence of non-neoplastic OGCs [6]. There have been a lot of controversies regarding the origin of OGCs. Epithelial, histiocytes or mesenchymal metaplasia have been proposed origins of OGCs. But given nuclear features, lack of reactivity with epithelial markers, cluster of differentiation (CD) 68 and lysozyme reactivity, most likely they originate from histiocytes. These characteristics of OGCs have been proposed to arise from mononuclear histiocytes/macrophages attracted by growth or chemotactic factors produced by the cancerous cells [2].

UC-OGCs are most commonly found in the body or tail of the pancreas. Multiple studies have shown the incidence of tumor in pancreatic body and tail to be about 70%. Hemorrhagic necrosis is common, occasionally calcifications can be seen and margins are usually clear. Distant metastases can occur in an advanced stage and most commonly it involves liver, lung, and bone [4].

The UC-OGC of the pancreas needs to be differentiated from pancreatic mucinous cystic neoplasm (MCT), solid pseudo-papillary neoplasm (SPN) and intraductal papillary mucinous neoplasm (IPMN). MCT is more common in elderly female and also found in the pancreatic tail. It presents as large cystic cavities, thick septa, and nodular protuberances in the mass. Histopathology shows ovarian-type stroma. SPN is more common in young women and presents as a solid-based, soft texture mass without pancreatic duct expansion. SPN is a borderline tumor with good prognosis, and local infiltration or distant metastases are extremely rare. IPMN is more common in older men and often occurs in the head of the pancreas and may cause jaundice [4]. 

En-bloc resection is usually the first line treatment but most studies have shown disappointing results with this rare tumor after surgery. Most of the case reports have shown that patients had early recurrence even after complete surgical removal and passed away within one-year post-surgery. There is limited data on the literature review regarding the role of adjuvant chemotherapy and radiotherapy, given the rarity of this neoplasm [1]. Due to the epithelial origin of the tumor, there are few case reports which have shown some favorable response to chemotherapeutic agents such as cisplatin, paclitaxel, and gemcitabine [7]. We started our patient on dual chemotherapy with gemcitabine and capecitabine but she did not tolerate it, now she is only receiving Gemcitabine. Based on radiosensitive nature of giant cell tumor of the bones, the role of radiotherapy in UC-OGC has been mentioned in the literature. However, given the lack of clear-cut objective and clinical data, no safe conclusions can be drawn.

Literature review

In a review of English literature, we conducted a literature search using PubMed from January 2010 to April 2019. The combination of keywords used were ("pancreatic") and ("osteoclast-like giant cell tumor or cancer or malignancy"). We found 15 cases in 12 reports. A summary of 15 cases of UC-OGC is shown in Table 1.

**Table 1:** Characteristics, treatment, and prognosis of osteoclast-like giant cell tumor of pancreas in included patients

Case Number.	Primary Author	Year of Publication	Age	Gender	Location of Cancer	Treatment	Prognosis
1.	Yepuri et al. [8]	2018	78	Male	Head of pancreas	Sub-total pancreatectomy with splenectomy	Alive three years post-surgery without recurrence
2.	Chen et al. [9]	2016	75	Female	Head of pancreas	Pancreaticoduodenectomy and lymph node dissection	Not reported
3.	Lahiff et al. Case 1 [10]	2016	77	Male	Head of pancreas	Whipple followed by adjuvant chemotherapy	Patient died in 14.5 months
4.	Lahiff et al. Case 2 [10]	2016	78	Female	Ampulla	Palliative by-pass surgery	Patient died in three months
5.	Lahiff et al. Case 3 [10]	2016	81	Male	Body of pancreas	None	Patient died in 11 months
6.	Lahiff et al. Case 4 [10]	2016	60	Female	Tail of pancreas	Distal pancreatectomy followed by adjuvant chemotherapy	Patient died in 12 months
7.	Georgiou et al. [11]	2015	75	Female	Head of Pancreas	Whipple procedure	Patient died in 10 months
8.	Sah et al. [12]	2015	54	Female	Body and tail of pancreas	Distal pancreatectomy with splenectomy	Not reported
9.	Gao et al. [6]	2015	71	Female	Body and tail of pancreas	Distal pancreatectomy with splenectomy followed by adjuvant chemotherapy	Alive 10 years post surgery
10.	Kobayashi et al. [13]	2014	37	Female	Head of pancreas	Pancreaticoduodenectomy	Patient died 66 months after surgery because of recurrence
11.	Temesgen et al. [1]	2014	57	Female	Tail of pancreas	Distal pancreatectomy, splenectomy and partial gastrectomy	Not reported
12.	Sungho jo [2]	2014	67	Female	Body and tail of pancreas	Sub-total pancreatectomy with splenectomy, total gastrectomy and segmental resection of transverse colon	Patient died nine months after surgery
13.	Wada et al. [14]	2011	59	Male	Tail of pancreas	Distal pancreatectomy with splenectomy, total gastrectomy and cholecystectomy	Patient died four months after surgery
14.	Rustagi et al. [5]	2011	62	Male	Head of pancreas	Whipple’s procedure	Not reported
15.	Mannan et al. [15]	2010	40	Female	Head and tail of pancreas	Pancreatico-jejunectomy	Not reported

10 patients were female and five patients were male. Seven tumors were located in the head of pancreas, one tumor in ampulla and seven tumors were located in body and/or tail of the pancreas. The mean age at diagnosis was 59 years (range 37-81 years). The 14/15 cases underwent surgical resection while one patient denied surgical intervention. The 3/15 cases underwent adjuvant chemotherapy. We found in our literature review, seven cases had mean survival rate eight months, while in five cases outcome was not mentioned. UC-OGC has worse prognosis as compared to invasive ductal adenocarcinoma of the pancreas. Median survival is 11 months in operable tumors while there is a significant drop in survival rate to six months in cases which are considered non-operable. According to Kobayashi et al., [13] younger age at the time of diagnosis, female gender, smaller lesions and absence of lymph node involvement are good prognostic factors. OGCs have better prognosis as compared to other pancreatic giant cell tumors. The case reported by Kobayashi et al. survived for almost five years after surgical intervention.

## Conclusions

Pancreatic osteoclast-like giant cell tumor is a very rare type of pancreatic cancer with unique histopathological characteristics. En bloc resection is usually the first line of treatment. Given the rarity of this tumor, the role of adjuvant or neo-adjuvant chemotherapy or radiotherapy, has not been clearly established. Few reports have shown beneficial response to adjuvant chemotherapy. However, given the lack of clear-cut objective and clinical data, no safe conclusions can be drawn regarding the actual benefit of therapy. In clinical practice, most patients are given a choice of therapy given the poor prognosis, with a clear explanation that clear-cut benefit has not been established in randomized clinical trials.
